# Nonclassical light from a large number of independent single-photon emitters

**DOI:** 10.1038/srep19760

**Published:** 2016-01-27

**Authors:** Lukáš Lachman, Lukáš Slodička, Radim Filip

**Affiliations:** 1 Department of Optics, Palacký University, 17. listopadu 1192/12, 771 46 Olomouc, Czech Republic

## Abstract

Nonclassical quantum effects gradually reach domains of physics of large systems previously considered as purely classical. We derive a hierarchy of operational criteria suitable for a reliable detection of nonclassicality of light from an arbitrarily large ensemble of independent single-photon emitters. We show, that such large ensemble can always emit nonclassical light without any phase reference and under realistic experimental conditions including incoherent background noise. The nonclassical light from the large ensemble of the emitters can be witnessed much better than light coming from a single or a few emitters.

A stark contrast between classical and quantum physics continuously attracts high attention of both theorists and experimentalists. The strict and large difference in the theoretical concepts of classical and quantum physics suggests that no smooth transition can exist^1^. On the other hand, continuous and fuzzy transitions between quantum and classical effects in many experiments provoke ongoing discussions about an existence of quantum effects in the domain of classical physics. The most publicly known example is the Schrödinger cat experiment^2^,^3^,^4^, which has stimulated many challenging experimental tests of quantum effects with a large number of particles. Also, non-classical features of light were tested on very strong squeezed states^5^. Further, in the series of experiments in trapped ions^6^,^7^, cavity-QED experiments^8^,^9^, at optical frequencies^10^,^11^, microwave frequencies^12^ and atomic ensembles^13^, the quantum correlation between a two-level system and an oscillator has allowed to prepare a nonclassical quantum state of a larger number of particles. The non-classicality means here an incompatibility with oscillations possible in classical coherence theory^14^. There have been developed many criteria of non-classicality, for example^15^,^16^, etc. However, as the number of particles increases, many already demonstrated nonclassical effects are critically sensitive to a coupling with an environment^17^. It seems to be therefore very challenging to observe the nonclassical effects at a truly macroscopic limit of large ensembles, which correspond to a traditional domain of classical physics.

Quantum optics may contribute significantly to this challenge. Essentially, any macroscopic state of light is generated by a large ensemble of emitters with discrete energy levels producing individual photons. The most basic are single photon emitters, developed recently with very small multi-photon contributions, as was demonstrated for two-level atoms^18^, trapped ions^19^, quantum dots^20^, NV centers^21^ or molecules^22^. Single photon states of light are advantageously insensitive to fragile phase and provably exhibit very robust nonclassical and even quantum non-Gaussian features^23^,^24^,^25^,^26^. They remain detectable for large optical losses, therefore suitable for a wide range of applications. Moreover, that robust non-classicality and quantum non-Gaussianity of light from a single emitter can be detected by a single-photon version of the Hanbury-Brown-Twiss (HBT) measurement^27^,^28^. Recently, an extension of this measurement has been used to derive a hierarchy of operational and reliable non-classicality criteria^29^.

In this paper we predict that arbitrary large ensemble of independent, incoherent and even low-efficiency single photon emitters can produce experimentally provable nonclassical light without any phase reference and under realistic conditions. To prove it, we modify the hierarchy of non-classicality criteria^29^ for light from many emitters. The modified criteria allow us to prove that if single-photon emitters individually produce nonclassical light testable by the criteria, then source consisting of an arbitrarily large number of these single photon emitters generates also nonclassical light. The proposed observation of nonclassical light is very robust against many imperfections of the source. We verify two positive aspects of emission from ensemble of single photon sources compared to emission from a single one. First, the non-classicality can be witnessed more reliably. Second, non-classicality of a larger number of emitters is more robust against the background noise. We suggest a feasible experimental test of non-classicality of light emitted by large number of trapped ions capable of verifying our theoretical predictions.

## Non-classicality criteria for many emitters

Nonclassical states of light are from the point of view of coherence theory defined as quantum states beyond the mixtures of coherent states^14^. A technique for their detection and corresponding applied criteria have to be able to reliably and unambiguously detect weakly nonclassical states of light without phase reference and without any prior assumption about their source. The detectors must be free of any systematic errors which could cause an overestimation of the non-classicality. Moreover, the criteria used for the tests of non-classicality have to be applicable also for a large number of emitters.

**Figure 1:**
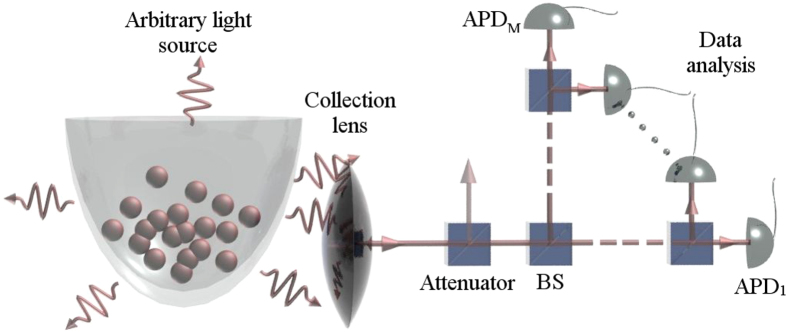
The proposed scheme for detection of nonclassicality. The light emitted from many emitters is partially collimated on a network of 50:50 beam-splitters (BS). The non-classicality can be detected by *M* avalanche photodiodes (APD), each in one of the output modes of the network. Probabilities *P*_0_ and *P*_0⊗*M*_ are determined from the registered detection events.

The methodology of such operational non-classicality criteria constructed purely from a prior knowledge about the detection scheme presented in the ref. 29 can be modified to our purpose. The criteria can be applied to an extension of Hanbury-Brown-Twiss type of the experiment with single photon detectors depicted in Fig. 1. The emitted light from the source impacts on a network of beam splitters (BS) and emerging beams are measured by avalanche photodiodes (APDs). They can distinguish typically with a low efficiency only photons from the vacuum. Both the inefficiency and dark counts of the APDs reduce the nonclassical aspects, but they cannot produce them. Ignoring them in the construction of the criteria will not therefore cause overestimation of the non-classicality. On the other hand, the detection technique needs to be free of any saturation effects which can artificially increase the estimated non-classicality. For the same reason, it is important to measure the effective splitting ratios including the transmissivity and reflectivity of the beam splitters together with the generally different efficiencies of the APDs and consider it for the derivation of the criterion.

The non-classicality can be proven from the hierarchy of linear functionals 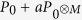 of the density matrix, in which probability *P*_0_ corresponds to events when a single chosen APD does not click and 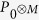 is the probability that no click is registered by all *M* APDs. Advantageously, we use a parametrization by *P*_0_ and 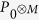, different to the one used in ref. 29, to be able to simply prove our results and reach the best recognition of nonclassical light. We consider now the network of beam-splitters splitting light symmetrically to all *M* detectors. Note that the proposed criteria can be modified for any unbalanced network, similarly to a simple example in ref. 29. Optimizing the functional 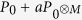 over all classical coherent states leads to a threshold function 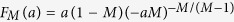, where 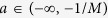. This limitation arises from an optimal mean number 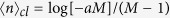 of photons in the classical coherent state. The linearity of the employed functional guarantees that any convex combination of coherent states cannot surpass the threshold *F*_*M*_(*a*) for any parameter *a*. In turn, the existence of *a* for which 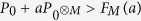 guarantees the non-classicality of a given state. The optimal choice of the parameter *a* for a particular probability 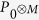 corresponds to minimizing right side of inequation 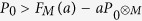 over *a*. It gives raise to an explicit form of sufficient conditions of non-classicality


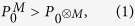


which cannot be fulfilled by any classical state of light. The coherent states reach 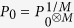. The condition (1) for *M* = 2 approximates the requirement yielded from commonly used measurement of intensity correlation function *g*^(2)^(0) for states with very low photon flux 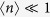
27. The derivation of the criterion (1) is exact, without any approximative steps used for example in refs 27,28. It is therefore valid even for bright sources of light, where the photon flux can not be substituted by a probability of a click. The proposed criteria (1) are based only on the knowledge about the detection method, for example the effective beam splitting ratio. It is therefore suitable to reliably detect very small non-classicality of bright light. Importantly, in equation (1) cannot be satisfied by any multi-mode classical state


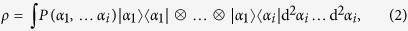


which is a consequence of the fact, that the threshold function *F*_*M*_(*a*) resulting from the optimization does not increase due to the multi-mode structure. The non-classicality of light from any source can be thus examined even for an arbitrary large number of modes.

Suitable form of the hierarchy of non-classicality criteria (1) allows to straightforwardly prove following result. Consider an ensemble of independent emitters. Each emitter gives raise to probability of no detection events *P*_0,*i*_ and 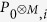, where subscript *i* denotes probabilities for an emitter *i*. Because emitters are considered independent, the vacuum probability of the whole ensemble yields 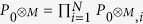 and 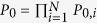. There is a connection between non-classicality of a single emitter and the non-classicality of whole ensemble following from the statement


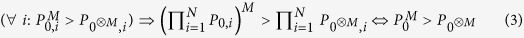


for arbitrary *M* ≥ 2, where 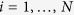. Therefore, any ensemble of independent emitters all individually satisfying (1) generates arbitrarily bright nonclassical light detectable by the same criterion (1). Due to a very suitable parametrization of the problem, the expression can be proved by simple algebraic rules. A main task was to find the most suitable parametrization of non-classicality criteria, so that the proof is simple and broadly understandable. Although participating *N* single-photon emitters can be quite different, the non-classicality of light emitted by each emitter suffices to obtain a nonclassical state yielded from arbitrarily large ensemble of them. In the Supplementary Material, we discuss a tolerance of the nonclassical light emission from realistically unstable large ensembles.

## Reliability of nonclassical criteria

To obtain a conclusive proof of non-classicality, the statistical errors of the measured distance from the non-classicality threshold have to be sufficiently small. The necessity of small errors dictates in turn a minimal time needed for such experimental estimation. We have chosen a logarithmic scale for its better interpretations. The criteria (1) get a linear condition 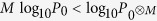. We consider a simplest example of an ensemble of *N* single photon emitters, where any emitter produces maximally a single photon in the state 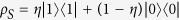. The probabilities for the criterion (1) are 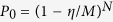 and 
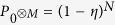
. Let us define distances along the axes for states above the threshold


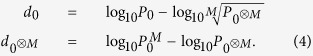


**Figure 2:**
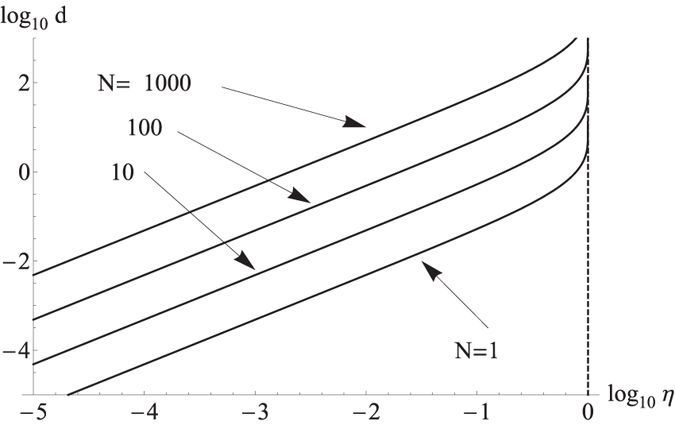
The reliability of the simplest criterion with *M* = 2 detectors in (1) is depicted for the discussed model 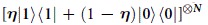. The reliability is qualified by the proposed distance *d* of a point 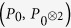 from the threshold using equation (5). It affirms the advantage of using a large ensemble of single photon states.

The convenient choice of the log-log space for the description of the distance from the threshold (4) connects both distances 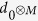 and *d*_0_ so that 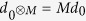. From the point of view of measurement, the relevant distance *d* from the threshold is given by 
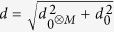
, which implies 
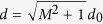
. If the state does not satisfy the condition of non-classicality (1), we assign zero to both distances 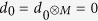 to avoid obtaining negative distance. We stress, that this distance does not quantify an amount of non-classicality. The distance for *N* emitters producing individually *ρ*_*S*_ with the same efficiency of generation *η* is


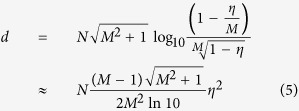


where the approximation is valid for typical low-efficient emitters with 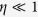. *Interestingly, light from many emitters increases the distance of the point from the non-classicality threshold*. As can be seen in Fig. 2, the distance is very sensitive to the single photon emitter efficiency *η*. Furthermore, the presented plot suggests, that small number of the experimental runs will suffice to obtain reliable estimate about non-classicality of emitted light. For larger number of detectors *M*, the distance *d* saturates to 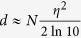 for small 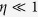. The small *η*^2^ can be advantageously compensated by a large number of emitters *N*. This opens possibility to detect non-classicality using only a fraction of emitted light.

## Background noise

Since 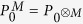 for the classical noise with Poissonian statistic, any source of multi-mode Poissonian noise added to the emitters of nonclassical light keeps non-classicality observable by (1), what can be simply proven using (3). On the other hand, thermal incoherent background noise limits detection of nonclassical light^25^. The influence of the thermal noise on light from *N* single photon emitters can be described by two principal models


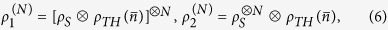


where 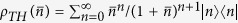 is thermal noise with a mean photon number of 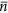. We investigated all criteria (1) and numerically verified that *the basic criterion for M* = 2 *is sufficient* for both 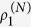 and 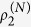 for arbitrary large *N*. It demonstrates that detection setup for nonclassical light from the large ensemble can be very simple. In many cases, we can use the simplest case with *M* = 2, therefore we will further focus on this simplest case.

In the state 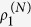, which corresponds to the noise contributing to each single emitter, non-classicality appears if 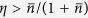. This condition holds for arbitrary large number of emitters. On the other hand, for the right state 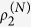, which describes the background noise jointly affecting all emitters, the threshold for non-classicality interestingly decreases for a large number of emitters *N*. For very low noise 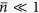, the nonclassical light is detected if


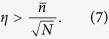


It is a clear application of many emitters, they can be used to *detect non-classicality not measurable from a single emitter*. For the state 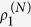, non-classicality tolerates optical attenuation when 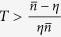, irrespectively to *N*. For 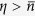, the non-classicality cannot be destroyed by attenuation and it is absolutely robust. For the state in 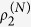, the approximate condition (7) appeared to be also for the absolute robustness.

## Measurement time

A remaining limit for the detection of nonclassical light seems to be only finite measurement time. The distance from the threshold (1) for *M* = 2 is depicted in the Fig. (2). The distances have to be smaller than demanded number of standard deviation units obtained by


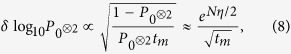


where *t*_*m*_ is measurement time. The approximation is correct only with assumption 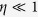 and 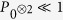. For a large number of emitters and corresponding large photon flux, the measurement time can be optimized by attenuation of photon flux. The attenuation with efficiency *T* modifies the probabilities to 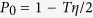 and 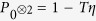. Clearly, any fixed number of these sources always radiates provably nonclassical light preserved through any attenuating channel. The attenuation can be optimized to reach sufficiently high 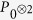 and simultaneously, not too small distance from the threshold. According to (5) and (8), it appropriates to optimization of the measured time 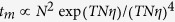. One can find an optimal photon flux *TNη* and the corresponding increase of time is quadratic in number of emitters. On the contrary, the necessary measurement time grows exponentially without performing the optimal attenuation. Since an arbitrarily large ensemble of such emitters generates non-classical light, its detection reduces to search of relevant experimental scheme and parameters optimization.

## Experimental proposal

**Figure 3:**
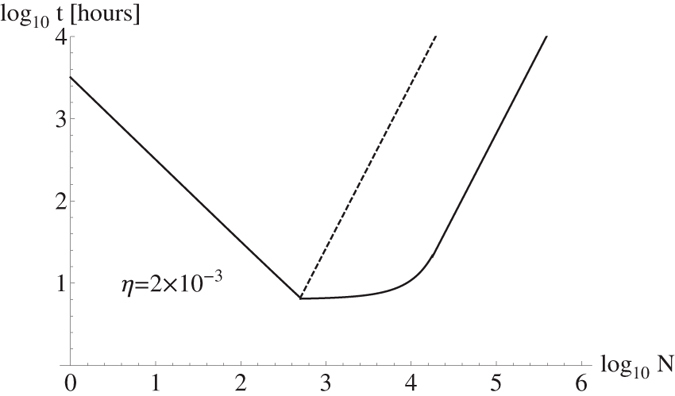
Estimation of the minimal time needed for the measurement of non-classicality of light from *N* trapped ions corresponding to reaching the distance *d* more than three standard deviations from the non-classicality threshold. The solid lines show simulation results employing attenuations of the measured signal using both periodic switching-off of the detectors and beam splitter attenuation, while the dashed lines corresponds to the case where detectors saturation is avoided only by more conventional beam-splitter method.

A large number of trapped atoms or ions^30^,^31^ can be advantageously used for an experimental demonstration. We consider a system containing *N* ions in a cigar-like shaped crystal^32^ trapped in a focus of a lens with a numerical aperture of 0.2 corresponding to 1% of the full solid angle being continuously driven by excitation laser. We estimate a minimal measurement time needed to proof non-classicality of emitted light, which we set to time needed for reaching *d*/*σ*_*d*_ = 3, where *σ*_*d*_ is one standard deviation of measured distance *d*. We assume that the number of ions in the trap is constant, which is justified if 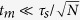, where *t*_*m*_ is measurement time, *τ*_*s*_ is storage time of an ion in the trap and *N* is a number of ions. Thus for very conservative storage time *τ*_*s*_ = 1 day and an ensemble containing several tens of ions, the measurement should no exceed one hour. If the required measurement time exceeds this assumption, the experiment can to be repeated several times with shorter time duration and with same initial number of emitters, so that statistics of decaying number of emitters is included in the measured vacuum probability. Collected photons are collimated and directed towards two APDs through variable attenuator and an ordinary 50:50 BS as depicted in Fig. 1. The emitted fluorescence collected by the lens is then detected with very modest overall detection efficiency *η* = 0.2%, which corresponds to realistic value of single photon detector quantum efficiency of 50%, 50:50 splitting ratio of the beam splitter and additional 20% absorbtion and reflection losses at optical elements. Considering the excitation and detection of the photons from the dipole transition of some frequently used alkaline earth metal ions with the excited state lifetime *t*_*e*_ ~ 100 ns, we set the length of the single measurement time-bin to 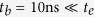 to minimize the multi-photon contributions from the same ion in the single time-bin. The efficiency *η* can be further artificially decreased by the variable attenuation with factor *T*. This attenuation strongly influences the measurement duration and one can find optimal *T* < 1 which minimizes the measurement time. The estimated optimal photon flux per single time bin in our case corresponds to *NTη*  ≈ 4.1. The probability of a click for such flux is 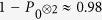. As can be seen in the simulation results presented in Fig. 3, our approach enables to demonstrate non-classicality of emitted light within a 100 hours for an ensemble of up to *N* ~ 10^4^ ions, which approaches a truly macroscopic limit.

In the presented simulation we account for the effect of saturation of conventional single photon detectors which becomes substantial typically at a count-rate of about 500 kHz, what corresponds approximately to *ηN* ~ 0.05. For the photon flux of the source below this value, the APDs can be opened per each temporal mode without being saturated. As can be seen in the Fig. 3, the minimal measurement time in this region rapidly decreases due to the simultaneous increase of the distance *d* and measured photon flux as the number of emitters *N* grows. Above this region, we start avoiding the saturation of detectors by keeping both APDs switched-off most of the time and opening them only for 10 ns ~ *t*_*b*_ periods with a frequency of the opening given by the saturation limit. Activation of this mechanism is responsible for a strong kink in the dependence of the minimal measurement time on the number of particles at *ηN* ~ 0.05. Further increase of the number of particles is compensated by decreasing the overall time in which APDs are switched on. The minimal measurement time in this region stays approximately constant up to the point, where the frequency of APD switching-off reaches 500 kHz, which corresponds to regime where one photon is detected at each APD in almost each detection period. We note, that we don’t consider a detection of more than one photon by single APD in a given detection interval, which is prohibited by dead time *η*_DT_ of APDs being typically 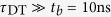. This assures, that further increase of number of emitters will not cause saturation in this regime. When compared to conventional beam-splitter type of attenuation to obtain the regime below detector’s saturation which is depicted by a dashed line in Fig. 3, this approach substantially reduces total measurement time. This is mainly caused by the fact, that an estimated optimal value of the *ηN* ≈ 4.1 is far behind the saturation limit of conventional single photon detectors. Further increase of the number of emitters and corresponding photon flux behind this value is then compensated by usual beam-splitter type of attenuation with attenuation factor *T* which gives a quadratic increase of measurement time.

## Application and outlook

Practically, our methodology can be generally applied as an efficient tool enabling unambiguous searching for nonclassical behavior hidden in various experimental platforms without any prior knowledge. A potential of nonclassical states of large ensemble can be further found in quantum metrology and quantum communication. The proposed experiment will likely move forward ongoing investigation of quantum to classical transitions with large quantum or even mesoscopic systems.

## Methods

### Statistics of emitters

Statistics of the number of emitters clearly influences the non-classicality of emitted radiation. If the emitters generate exactly single photon, the thermal (Poissonian) statistics of emitters will produce thermal (Poissonian) statistics of light. On the other hand, the emitters producing states 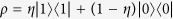 can be measured as nonclassical using the hierarchy of criteria (Eq. (1) in the main text) only if
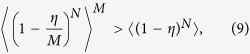
where the averaging is over the fluctuating number of emitters. Inserting low efficiency approximation 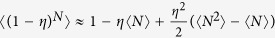  and 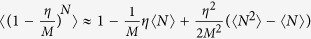 with 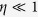 into the hierarchy of criteria (Eq. (1) in the main text) and considering terms only up to *η*^2^, we obtain that the variance


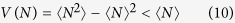


has to be smaller than Poissonian to obtain the nonclassical light detectable for any *M* ≥ 2.

The approximation of small 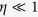 for (10) has been verified numerically and compared to (9). Sub-Poissonian statistics of single photon emitters therefore suffices to generate detectable nonclassical light. For the simplest scheme with *M* = 2 and the ensemble of imperfect single-photon emitters producing an approximative state 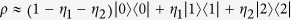, where 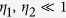 are small single-photon and two-photon contributions, we can approximatively obtain the condition 
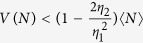
. The ratio 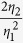 corresponds to the ratio 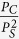 converging to the correlation function *g*^2^(0) for *η*_1_, 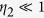, where 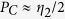 is a probability of a coincidence detection and 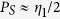 is approximately a probability of a detection event at the single detector. Non-vanishing *g*^2^(0) therefore suppresses the upper bound on *V*(*N*) below the Poissonian limit. If the number of emitters is controlled reasonably below Poissonian limit, the detection of nonclassical states from a large number of emitters is feasible.

### Decaying number of emitters

For the test of non-classicality of emitted light from *N* emitters, it is not needed to know the number of emitters. It is ideal for the test, however, the number of single photon emitters exponentially decays with relatively small time constant in many experimental platforms. They can either gradually leave the ensemble to the environment or they can gradually loose possibility to emit light. Let us consider an ensemble that initially contains *N* single photon emitters radiating maximally a single photon with probability *η*. Further, we assume that a probability that an emitter radiates in the ensemble at time *t* is 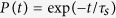, where *τ*_*s*_ is storage time of the emitter in the ensemble. The photon distribution of such source in time is


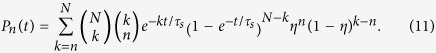


According to (11), it is possible to understand the losses of emitters from the ensemble as an attenuation of emitted light with the transmittancy 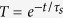. Thus, the fluctuating number of emitters can be included in the efficiency of the individual emitters 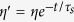, which enters the vacuum probabilities 
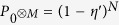
 and 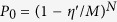. Since *η* → *η*′, all previous conclusions for *η* > 0 remain valid for *η*′ > 0, if the measurement starts in time *t*_0_ and lasts for time *t*_*m*_ much shorter than 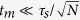 hence the number of emitters is stable during measurement. However, long measurement time or a large number of emitters violate this assumption and the detected vacuum probability has to be averaged


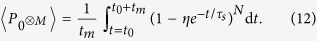


The average probability 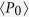 is yielded by substitution *η* → *η*/*M* in (12). The view, how this averaging limits the observation of non-classicality, is reached in an approximation of very weak sources 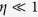, where the integration is analytically achievable. The first two members in Taylor expansion are 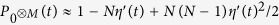 with 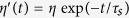. To expand *P*_0_(*t*), we can substitute *η* → *η*/*M* in the expansion of 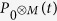. The subtraction 
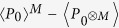
 cancels the parts proportional to *η* automatically. The second parts proportional to *η*^2^ do not contribute to the expansion only if 
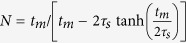
 for any *M* ≥ 2. Consequently, it dictates condition on a maximal number of single photon emitters such that the measurement duration *t*_*m*_ does not cause lost of non-classicality for 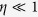 and any *M* ≥ 2


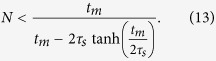


The small time *t*_*m*_ compared to the storage time gives approximative condition 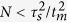. The lost of visibility of non-classicality can be avoided by repeating the measurement several times with the same initial number of emitters.

### Fluctuating single-photon efficiency

The efficiency of generation and detection of emitted single photons from individual emitters *η* can fluctuate during the measurement time, although the number of emitters is definite. The probability of vacuum is then 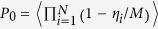 and 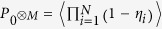, where *η*_*i*_ is the random efficiency of generation in *i*th emitter and 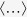 means mean value. Let all the emitters have 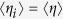. Considering that any two emitters do not influence each other, we use 
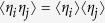
, 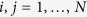 to obtain 
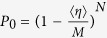
 and 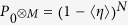. It clearly satisfies 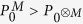. The different and fluctuating *η* of single photon emitters thus cannot hinder the observability of nonclassical light. We can detect non-classicality for the smallest *M* = 2, however, the distance from the threshold increases for larger *M*.

## Additional Information

**How to cite this article**: Lachman, L. *et al.* Nonclassical light from a large number of independent single-photon emitters. *Sci. Rep.* **6**, 19760; doi: 10.1038/srep19760 (2016).
